# Non-femoral head MRI radiomics as a potential imaging biomarker for identifying osteonecrosis of the femoral head in systemic lupus erythematosus patients

**DOI:** 10.3389/fmed.2026.1889377

**Published:** 2026-07-22

**Authors:** Yujing Zhang, Qing Hou, Shasha Zheng, Xinwen Wang, Xiaoli Song, Wenjin Bian, Jianing Cui, Jinliang Niu

**Affiliations:** 1 College of Medical Imaging, Shanxi Medical University, Department of Medical Imaging, Second hospital of Shanxi Medical University, Taiyuan, Shanxi, China; 2 Department of Radiotherapy, Shanxi Province Cancer Hospital, Shanxi Hospital Affiliated to Cancer Hospital, Chinese Academy of Medical Sciences, Cancer Hospital Affiliated to Shanxi Medical University, Taiyuan, Shanxi, China; 3 Department of Rheumatology and Immunology, Second hospital of Shanxi Medical University, Shanxi, China; 4 Department of Medical Imaging, Second hospital of Shanxi Medical University, Taiyuan, Shanxi, China; 5 College of Medical Imaging, Shanxi Medical University, Taiyuan, Shanxi, China

**Keywords:** identification, MRI, osteonecrosis, radiomics, systemic lupus erythematosus

## Abstract

**Purpose:**

To explore whether MRI-based radiomics features derived from non-femoral head bone marrow regions differ between symptomatic systemic lupus erythematosus (SLE) patients with and without osteonecrosis of the femoral head (ONFH), and to preliminarily evaluate their potential diagnostic utility.

**Methods:**

This retrospective study enrolled SLE patients from the primary institution (temporal split into training/internal validation cohorts) and an external institution using same-vendor scanners (external validation cohort). Radiomics features were extracted from the greater trochanter and ilium on T1-weighted images. A three-step feature selection strategy integrating LASSO, random forest (RF), and support vector machine recursive feature elimination (SVM-RFE) was applied to enhance feature robustness. Clinical, radiomics, and combined models were constructed and evaluated using ROC analysis, calibration curves, and decision curve analysis (DCA).

**Results:**

A total of 123 patients were included in the primary cohort and 44 in the external validation cohort. The combined model achieved AUCs of 0.937, 0.889, and 0.861 in the training, internal validation, and external validation cohorts, respectively. The combined model yielded numerically higher AUC than the clinical model across all datasets. The radiomics model alone demonstrated stable discrimination and favorable net benefit in internal and external validation, whereas adding clinical variables did not improve clinical utility. DCA shows that in the validation cohort, the independent radiomics model was more stable in terms of net clinical benefit compared to the combined model. The inter-group differences in the external cohort did not reach a statistically significant level, which might be attributed to insufficient sample size and type II errors.

**Conclusion:**

MRI-based radiomics features derived from non-femoral head bone marrow regions differ significantly between symptomatic SLE patients with and without ONFH, suggesting potential imaging biomarker for diagnostic differentiation. These exploratory findings warrant further validation in prospective studies.

## 1 Introduction

Systemic lupus erythematosus (SLE) is an autoimmune disease characterized by immune complex deposition and multisystem inflammatory involvement (1). A prospective MRI-based study has revealed that ischemic osteonecrosis can be found in about 40% of SLE patients (2). Specifically, approximately 20.5% of patients with SLE develop osteonecrosis of the femoral head (ONFH), with bilateral involvement being common (3, 4). Its early detection poses a significant diagnostic challenge due to the vague and non-specific clinical presentation (5). And, ONFH represents an irreversible form of organ damage that progressively leads to severe hip dysfunction and ultimately requires surgical intervention (6). Moreover, the treatment method of ONFH in SLE patients remains challenging (7). Therefore, early identification of patients at high risk of ONFH is crucial for timely intervention.

Several clinical factors, including glucocorticoid exposure, disease activity, antiphospholipid antibodies, and lipid metabolism abnormalities, have been associated with ONFH development in SLE (3, 8). Although clinical models based on these variables have been proposed, their performance remains modest, and many rely on laboratory or genetic markers that are not routinely available in clinical practice (9–12). Consequently, there remains a need for objective imaging biomarkers that can reflect disease-related skeletal alterations.

Multiple imaging modalities have been explored for the early detection or risk assessment of ONFH. For example, Dynamic contrast-enhanced MRI and single-photon emission computed tomography/computed tomography (SPECT/CT) enable qualitative and quantitative assessment of femoral head perfusion, proving useful in predicting ONFH among patients with femoral neck fractures (13–15). Proton magnetic resonance spectroscopy has the potential to detect the early changes of lipid/water composition in bone, which is helpful for the risk stratification of ONFH (16). However, all of them require special equipment and are not part of the routine hip joint MRI examination procedures. And, few imaging studies have focused specifically on ONFH in SLE populations.

Although in-depth investigation of the dynamic changes within the bone marrow microenvironment can provide valuable insights into the pathogenesis of osteoarticular involvement in patients with SLE, conventional magnetic resonance imaging techniques often fail to directly detect subtle alterations in the bone marrow microenvironment. Radiomics is an emerging technology that can non-invasively extract high-throughput quantitative features from medical images and identify imaging features that are not visually appreciable (17). These features can reflect underlying physiological and pathological tissue characteristics, so as to identify histological subtypes, predict metastasis and estimate treatment response or prognosis (18–20). Previous studies have also demonstrated that CT- and MRI-based radiomics can classify osteoporosis by analyzing trabecular microstructure and bone marrow composition (21, 22). MRI-derived trabecular and bone marrow features have been shown to identify bone fragility in patients with equivalent bone mineral density (23). Given its ability to detect early subchondral architectural changes in knee, mandibular condyles, and iliac crests, MRI-based radiomics serves as an imaging biomarker for distinguishing osteoarthritic and non-osteoarthritic joints (24, 25). Radiomics analysis may also be useful for discrimination of reversible and irreversible bone marrow lesions, so as to guide the treatment plan and prevent the occurrence of bone necrosis (26). Currently, the application scope of radiomics in osteonecrosis of the femoral head (ONFH) mainly involves the analysis of the femoral head, aiming to facilitate the early diagnosis of ONFH and predict femoral head collapse (27–29). Overall, this approach enables high-throughput extraction of quantitative imaging features and offers a non-invasive approach to detect subtle bone marrow changes. However, whether radiomics features from remote bone marrow regions contain clinically relevant information regarding ONFH remains largely unexplored.

In SLE, systemic immune dysregulation, chronic inflammation, and glucocorticoid exposure can lead to microvascular impairment and bone marrow remodeling (30–33). These alterations are not confined to the femoral head. Histological and metabolic studies have demonstrated bone marrow abnormalities in the iliac crest and proximal femur in patients with osteonecrosis (34–36). The greater trochanter and ilium contain abundant cancellous bone and active marrow, making them sensitive to such systemic skeletal changes. T1WI primarily reflects bone marrow fat/water composition, and texture-based radiomics features can quantify subtle microstructural heterogeneity. Based on this rationale, we hypothesized that radiomics features extracted from the greater trochanter and ilium may capture early, systemic marrow alterations associated with ONFH.

Therefore, this exploratory study aimed to compare radiomics features from the greater trochanter and ilium between symptomatic SLE patients with and without ONFH, and to assess whether these features may serve as imaging markers of bone marrow alterations associated with ONFH.

## 2 Materials and methods

### 2.1 Patient selection

This retrospective study was approved by the Institutional Review Board of the Second Hospital of Shanxi Medical University, with a waiver of informed consent. All procedures were conducted in accordance with the Declaration of Helsinki. Ethical approval was also obtained from Shanxi Bethune Hospital for the external validation cohort.

A total of 153 patients with systemic lupus erythematosus (SLE) who underwent hip MRI between February 2015 and December 2024 were initially identified. All patients met either the 2012 Systemic Lupus International Collaborating Clinics (SLICC) criteria or the 2019 European League Against Rheumatism/American College of Rheumatology (EULAR/ACR) classification criteria. Magnetic resonance images were independently evaluated by two musculoskeletal radiologists with extensive experience. According to the Association Research Circulation Osseous (ARCO) staging system, ONFH cases were classified as stage I–IIIA.

The inclusion criteria were as follows: (1) diagnosis of SLE for more than 6 months; (2) closed epiphyseal plates; and (3) presence of groin pain for more than 2 weeks with limited internal rotation on physical examination. The exclusion criteria were: (1) SLE duration < 6 months; (2) prior diagnosis or treatment of osteonecrosis; (3) malignant bone lesions, hematological diseases, or metabolic bone disorders; (4) femoral head collapse >2 mm or extensive bone marrow edema; (5) osteonecrosis or edema in the greater trochanter; and (6) poor image quality or incomplete imaging data. Exclusion of femoral head collapse >2 mm was based on ARCO stage IIIB (surgical threshold for irreversible joint damage); additionally, advanced collapse may alter hip biomechanics and secondarily affect iliac bone marrow composition, potentially confounding our remote-site radiomics analysis.

After applying the inclusion and exclusion criteria, 123 patients were eligible for the study. Patients were divided into a training cohort (*n* = 86) and a validation cohort (*n* = 37) using a temporal split (before and after December 2023).

In addition, an independent external validation cohort was established from Shanxi Bethune Hospital. Between 2020 and 2025, 44 consecutive patients with SLE who met the same inclusion and exclusion criteria were enrolled, including 19 patients with ONFH and 25 without ONFH.

### 2.2 Data collection

MRI data were retrieved from the Picture Archiving and Communication System (PACS). MRI examinations at the primary institution were performed using two 3.0T scanners (GE SIGNA Pioneer and GE Discovery MR750) with standardized imaging protocols. All patients were positioned supine with neutral lower limb alignment, and motion was minimized using fixation straps. A 16-channel body phased-array coil was used for hip imaging. In this retrospective study, T2-FS/STIR sequences were not consistently acquired across all patients, and their parameters varied considerably, which would compromise radiomics feature standardization. T1WI, which primarily reflects bone marrow fat/water composition, was available for all patients and showed higher inter-scanner reproducibility in our preliminary assessment. Therefore, only T1WI was used. Although minor differences existed between scanner models, consistent acquisition parameters were applied to ensure comparability.

The external validation cohort from Shanxi Bethune Hospital was acquired using a 3.0T MRI scanner from the same vendor (GE Healthcare). Similar imaging protocols was applied across institutions.

Demographic information (sex and age at SLE diagnosis) and laboratory parameters (coagulation indices and lipid profiles) were collected.

### 2.3 Segmentation of ROIs and extraction of radiomic features

**Figure 1:**
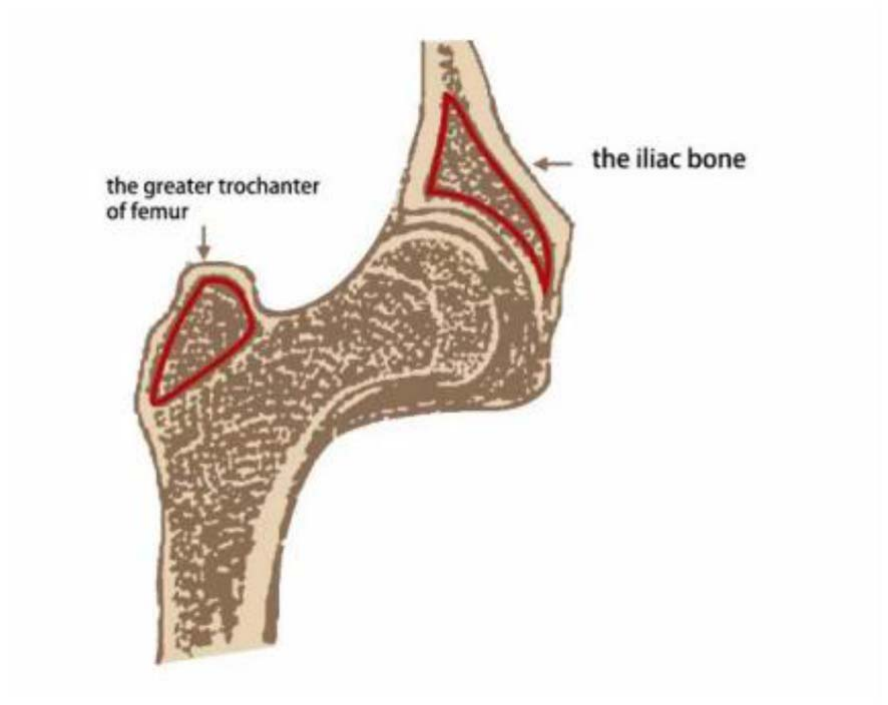
Definition of regions of interest (ROIs) in the greater trochanter and ilium on coronal T1-weighted MRI. ROIs were manually delineated on coronal T1-weighted images, excluding cortical bone. The greater trochanter ROI was confined within the epiphyseal line. The ilium ROI was defined with anterior and posterior boundaries corresponding to the acetabular margins, a superior boundary 2 cm above the femoral head apex, and an inferior boundary at the acetabular inferior margin.

Regions of interest (ROIs) were manually delineated on coronal T1-weighted images by two experienced musculoskeletal radiologists, with initial segmentation performed by one reader and subsequently reviewed by a second senior radiologist to reach consensus. Two ROIs were analyzed: the greater trochanter and the ilium (Figure 1), both excluding cortical bone. The greater trochanter ROI was confined within the epiphyseal line. The ilium ROI was defined with anterior and posterior boundaries at the acetabular margins, a superior boundary 2 cm above the femoral head apex, and an inferior boundary at the inferior acetabular margin. For patients with bilateral ONFH, MRI features were extracted from the hip with the more advanced ARCO stage (or the right hip if stages were identical). For patients without ONFH, one hip was randomly selected for analysis. Therefore, no patient contributed data from both hips.

All images were resampled to isotropic voxels (1 × 1 × 1 mm^3^) prior to feature extraction. Radiomics features were extracted using PyRadiomics (version 3.0.1) in accordance with the Image Biomarker Standardization Initiative (IBSI). Intensity normalization was performed using *z*-score standardization. Shape features were excluded due to variability in ROI size and geometry across patients.

Interobserver reproducibility was assessed in 30 randomly selected cases, and only features with intraclass correlation coefficients (ICC) >0.85 were retained for subsequent analysis.

### 2.4 Features selection

Both clinical and MRI radiomics data were analyzed. Clinical predictors were identified from 11 variables using univariate and multivariate logistic regression analyses.

For the feature selection in radiomics, we first applied univariate logistic regression to identify the candidate features. Subsequently, three complementary machine learning methods—LASSO, random forest (RF), and support vector machine recursive feature elimination (SVM-RFE)—were independently used for feature selection. No information from the internal or external validation cohorts was used during hyperparameter tuning. However, nested cross-validation was not performed due to the moderate sample size; this is acknowledged as a limitation. Only features consistently selected across multiple algorithms were retained. The rationale for taking the intersection of LASSO, random forest, and SVM-RFE was to retain features that are consistently selected by complementary algorithms, thereby reducing the risk of overfitting and improving cross-dataset stability compared with using a single method or the union of all selected features. Finally, multivariate logistic regression with Akaike Information Criterion (AIC)-based optimization was used to derive the final feature set and construct the radiomics score (Rad-score). All feature selection procedures were strictly performed in the training cohort to avoid data leakage. Although one radiomics feature (B-original_glr1m_ShortRunLowGrayLevelEmphasis) had a *P*-value of 0.058 in the final multivariable logistic regression model, feature selection in our study was not based solely on individual *P*-values. The model was developed using an information-theoretic approach based on Akaike's Information Criterion (AIC), rather than relying solely on univariate statistical significance. Under this framework, variable selection is driven by overall model fit and parsimony, not by a fixed *P*-value threshold. Therefore, variables with *P* > 0.05 may still be retained if their inclusion improves model performance or contributes to a better balance between goodness-of-fit and model complexity.

### 2.5 Model construction and assessment

Three models were constructed, including a clinical model, a radiomics model, and a combined model integrating both radiomics and clinical factors. Model development was performed exclusively in the training cohort. The performance of the developed models was then evaluated in the internal validation cohort and the independent external validation cohort without refitting. Model discrimination, calibration, and clinical utility were assessed using receiver operating characteristic (ROC) analysis, calibration curves, and decision curve analysis (DCA), respectively.

### 2.6 Statistical methods

All statistical analyses were performed using R software (version 4.5.1). Continuous variables were presented as mean ± standard deviation or median (interquartile range), as appropriate. Normality was assessed using the Shapiro–Wilk test. For comparisons between two groups (ONFH vs. non-ONFH), independent samples *t*-tests were used for normally distributed variables, and the Mann–Whitney *U*-test was used for non-normally distributed variables. Comparisons across cohorts were performed using one-way ANOVA or the Kruskal–Wallis test, as appropriate. Categorical variables were compared using the chi-square test or Fisher's exact test. Univariable and multivariable logistic regression analyses were performed to identify independent predictors, with variables showing *P* < 0.05 in univariable analysis entered into multivariable analysis.

Receiver operating characteristic (ROC) curves were generated using the pROC package, and areas under the curve (AUCs) were compared using DeLong's test. Calibration curves were constructed using the rms package with 1,000 bootstrap resampling. Decision curve analysis (DCA) was performed using the rmda package. A two-sided *P*-value < 0.05 was considered statistically significant.

## 3 Results

### 3.1 Clinical characteristics

A total of 123 patients from the primary institution were included, comprising 58 patients with ONFH and 65 without ONFH. These patients were temporally divided into a training cohort (*n* = 86) and an internal validation cohort (*n* = 37). An independent external validation cohort consisting of 44 patients from Shanxi Bethune Hospital (19 with ONFH and 25 without ONFH) was further included. In the primary cohort (*n* = 58 ONFH patients), the distribution was: Stage I 27.6%, Stage II 46.6%, Stage IIIA 25.8%. In the external validation cohort (*n* = 19 ONFH patients), 15 patients (78.9%) had ARCO stage I–II, and 4 patients (21.1%) had stage IIIA. Thus, advanced stages did not dominate, and the model performance was not driven by late-stage disease. Baseline clinical characteristics were largely comparable across the training, internal validation, and external validation cohorts, with no statistically significant differences observed for most variables (all *P* > 0.05; Table 1).

**Table 1:** Baseline clinical characteristics of patients in the training, internal validation, and external validation cohorts.

Variable	Overall (*N* = 167)	Train cohort (*N* = 86)	Internal validation cohort (*N* = 37)	External validation cohort (*N* = 44)	*P*-value	SMD
*n*	167	86	37	44		
TC (mmol/L) (median [IQR])	3.79 [3.40, 4.22]	3.88 [3.34, 4.24]	3.71 [3.44, 4.12]	3.73 [3.47, 4.30]	0.811	0.084
TG (mmol/L) (median [IQR])	1.80 [1.45, 2.16]	1.80 [1.35, 2.24]	1.78 [1.52, 2.11]	1.80 [1.56, 2.16]	0.952	0.090
HDL-C (mmol/L) (median [IQR])	1.22 [1.08, 1.33]	1.19 [1.09, 1.30]	1.25 [1.06, 1.33]	1.25 [1.10, 1.37]	0.372	0.142
LDL-C (mmol/L) (median [IQR])	1.90 [1.48, 2.20]	1.91 [1.47, 2.17]	1.91 [1.55, 2.23]	1.88 [1.63, 2.35]	0.562	0.184
PT (s) (median [IQR])	11.42 [10.14, 12.52]	11.37 [10.14, 12.49]	11.71 [10.42, 12.80]	11.40 [10.11, 12.46]	0.349	0.206
TT (s) (median [IQR])	17.40 [16.82, 18.17]	17.56 [16.82, 18.25]	17.36 [16.80, 18.07]	17.36 [16.97, 18.11]	0.900	0.060
D-dimer (μg/ml) (median [IQR])	1.49 [0.56, 2.04]	1.46 [0.57, 1.91]	1.58 [0.63, 2.08]	1.50 [0.42, 2.12]	0.397	0.131
APTT (s) (median [IQR])	28.73 [25.75, 31.18]	29.00 [25.25, 31.57]	28.71 [26.41, 32.05]	28.34 [26.08, 29.92]	0.619	0.200
FIB (g/L) (median [IQR])	3.55 [3.07, 4.09]	3.56[3.08, 4.04]	3.49 [3.06, 4.06]	3.50 [3.06, 4.24]	0.741	0.115
Gender (%)					0.913	0.054
Man	16 (9.6)	9 (10.5)	3 (8.1)	4 (9.1)		
Woman	151 (90.4)	77 (89.5)	34 (91.9)	40 (90.9)		
Age of SLE diagnosis (year) (median [IQR])	30.63 [25.59, 37.33]	30.61 [21.17, 36.31]	30.69 [27.02, 37.51]	30.88 [24.61, 38.76]	0.761	0.125

Data are expressed as medians (quartile ranges), mean (standard deviation) or numbers (percentages). ONFH, osteonecrosis of the femoral head; TC, total cholesterol; TG, triglyceride; LDL, low-density lipoprotein; HDL, high-density lipoprotein; PT, prothrombin time; TT, thrombin time; APTT, activated partial thromboplastin time; FIB, fibrinogen. Most SMD values were < 0.2, indicating good balance; one variable (PT) had an SMD of 0.206, suggesting a minor imbalance that is unlikely to affect model performance given the exploratory nature of the study.

Among the evaluated clinical variables, triglycerides (TG) and HDL-C were identified as independent factors of ONFH in the training cohort based on multivariable logistic regression analysis (Table 2).

**Table 2:** Univariable and multivariable logistic regression analyses of clinical factors for ONFH in the training cohort.

Variable	Univariable analysis			Multivariable analysis		
	*OR*	95% *CI*	*P*	*OR*	95% *CI*	*P*
TC	1.06	0.87–1.30	0.561			
TG	1.29	1.08–1.53	0.005	1.24	1.04–1.47	0.016
HDL_C	0.44	0.24–0.82	0.011	0.51	0.24–0.82	0.034
LDL	0.92	0.74–1.15	0.478			
PT	1.02	0.94–1.10	0.683			
TT	1.04	0.92–1.17	0.522			
D-Dimer	1.03	0.89–1.19	0.725			
APTT	1.01	0.98–1.04	0.561			
FIB	0.95	0.82–1.11	0.545			
Gender	0.73	0.52-1.03	0.080			
Age of SLE diagnosis	1.00	0.99–1.02	0.792			

TC, total cholesterol; TG, triglycerides; LDL, low-density lipoprotein; HDL, high-density lipoprotein; PT, prothrombin time; TT, thrombin time; APTT, activated partial thromboplastin time; FIB, fibrinogen.

### 3.2 MRI radiomics features

A total of 1,023 radiomics features were extracted from each region of interest (ROI) for every patient, including 18 first-order features, 24 gray-level co-occurrence matrix (GLCM) features, 16 gray-level run-length matrix (GLRLM) features, 16 gray-level size zone matrix (GLSZM) features,14 gray-level dependence matrix (GLDM) features, and 5 neighbor gray-level difference matrix (NGTDM) features. In addition, 930 high-dimensional features were extracted, comprising 744 wavelet-transformed features and 186 Laplacian of Gaussian (LoG) filtered features.

The distribution of ICC values for all extracted features is shown in Supplementary Figure S1. After filtering, 560 features from the ilium and 499 features from the greater trochanter with ICC >0.85 were retained for subsequent analysis.

**Figure 2:**
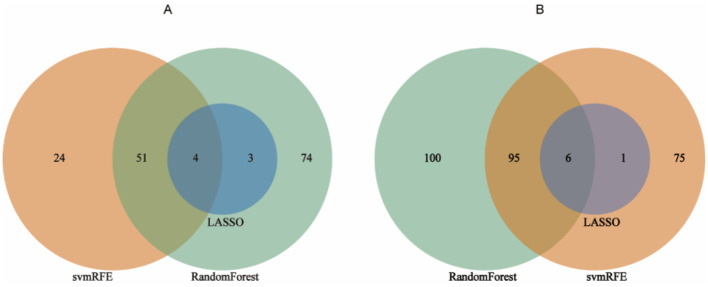
Radiomics feature selection workflow based on a multi-algorithm consensus strategy. **(A)** Greater trochanter: LASSO selected 7 features, RF selected 132, and SVM-RFE selected 79; 4 features overlapped across all three methods. **(B)** Ilium: LASSO selected 7 features, RF selected 201, and SVM-RFE selected 177; 6 features overlapped (LASSO, least absolute shrinkage and selection operator; RF, random forest; SVM-RFE, support vector machine recursive feature elimination).

**Figure 3:**
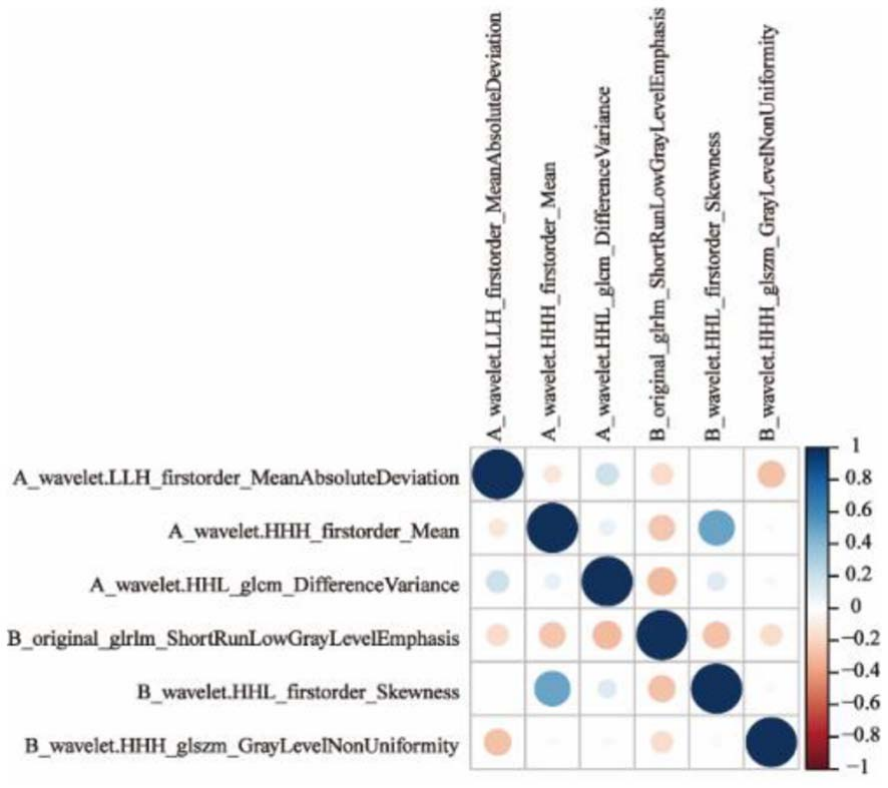
Correlation heatmap of the selected radiomics features. The heatmap shows pairwise correlations among selected radiomics features from the greater trochanter and ilium. No strong correlations were observed (|*r*| < 0.7), indicating low redundancy among selected features.

Feature selection was performed through a three-step process. First, a total of 178 and 382 radiomics features were identified by univariate logistic regression from the greater trochanter and the ilium, respectively. Subsequently, by three machine learning algorithms, four and six overlapping features were identified in the greater trochanter and ilium, respectively (Figures 2A, B). Finally, three significant features were extracted from each region of interest by multivariate logistic regression analysis based on minimum AIC (Supplementary Table S1). Correlation analysis revealed no significant correlation between features from the two ROIs (Figure 3). A radiomics score was calculated by weighting each selected feature by its coefficient.

### 3.3 Model construction and comparison

The final selected imaging features were used to construct the radiomics model. The clinical model was constructed based on independent factors identified from multivariable logistic regression analysis, namely triglycerides (TG) and HDL-C. The combined model integrated clinical and radiomics factors. Model performance was evaluated using ROC analysis.

**Figure 4:**
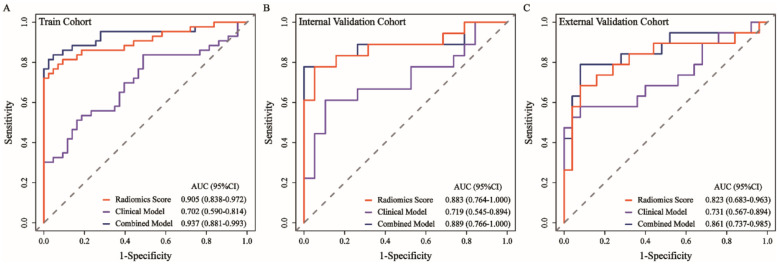
Receiver operating characteristic (ROC) curves of clinical, radiomics, and combined models. **(A)** Training cohort; **(B)** internal validation cohort; **(C)** external validation cohort. The combined model demonstrated numerically higher discrimination compared with the clinical model, and comparable performance to the radiomics model, with stable results across cohorts.

In the training cohort, the AUCs of the radiomics, clinical, and combined models were 0.905, 0.702, and 0.937, respectively. In the internal validation cohort, the corresponding AUCs were 0.883, 0.719 and 0.889. In the external validation cohort, the AUCs were 0.823, 0.731, and 0.861, respectively. Table 3 summarizes the sensitivity, specificity, PPV, and NPV for all three models across the three cohorts. Across all cohorts, the combined model showed numerically higher discrimination compared with clinical models in the training and internal validation cohorts, with stable performance observed in the independent external validation cohort (Figure 4).

**Table 3:** Performance metrics of the clinical, radiomics, and combined models.

Model	Cohort	Sensitivity	Specificity	PPV	NPV
Clinical	Training	0.837	0.512	0.632	0.759
Clinical	Internal	0.611	0.895	0.846	0.708
Clinical	External	0.579	0.920	0.846	0.742
Radiomics	Training	0.814	0.907	0.897	0.830
Radiomics	Internal	0.778	0.947	0.933	0.818
Radiomics	External	0.684	0.920	0.867	0.793
Combined	Training	0.837	0.953	0.947	0.854
Combined	Internal	0.778	1.000	1.000	0.826
Combined	External	0.789	0.920	0.882	0.852

DeLong test indicated that the combined model significantly outperformed the clinical model in the training and internal validation cohorts (*P* < 0.05) and was comparable to the radiomics model. In the external cohort, the combined model showed a numerically higher AUC (0.861) compared with the clinical model (0.731), but the difference did not reach statistical significance (DeLong test, *P* = 0.123). *Post-hoc* power analysis revealed that to detect this AUC difference with 80% power, approximately 102 subjects (44 ONFH cases and 58 non-ONFH controls) would be required, whereas the external cohort included only 44 subjects (19 ONFH cases and 25 non-ONFH controls). Therefore, the non-significant finding is likely attributable to Type II error (false negative) due to insufficient power.

### 3.4 Clinical implementation of combined model

**Figure 5:**
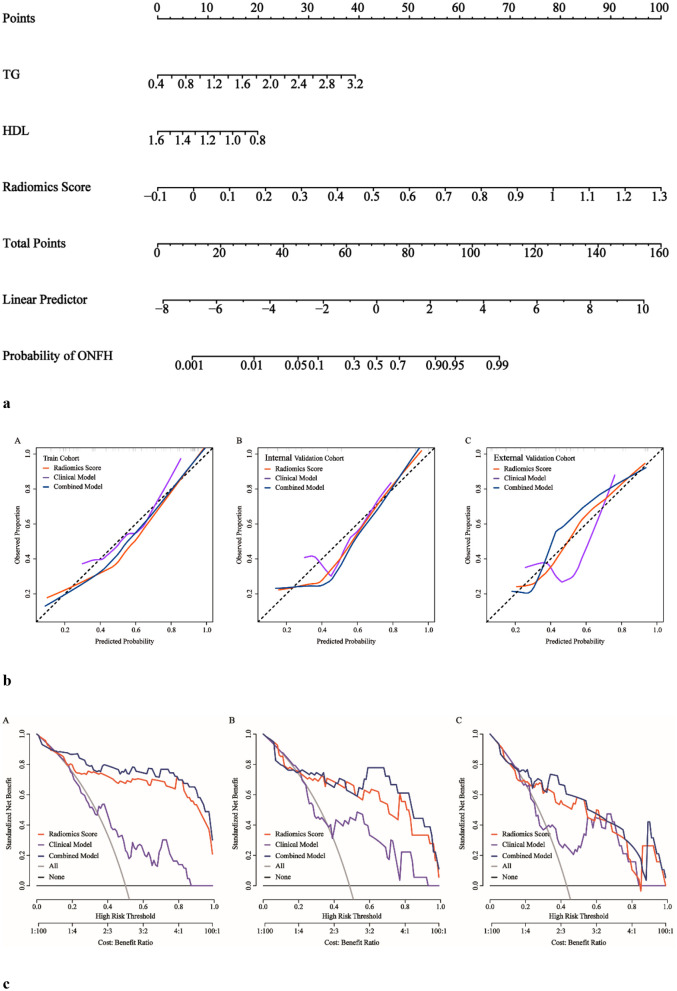
Nomogram, calibration curves, and decision curve analysis of the combined model for identifying ONFH in SLE patients. **(a)** Nomogram for identifying ONFH in SLE patients. **(b)** Calibration curves demonstrating agreement between estimated and observed probabilities in the training, internal validation, and external validation cohorts. **(c)** Decision curve analysis (DCA) showing net clinical benefit across threshold probabilities in the training, internal validation, and external validation cohorts.

The combined model was visualized as a nomogram (Figure 5a), incorporating TG, HDL-C, and Rad-score to provide an individualized probability estimation for the presence of ONFH. Each variable was assigned a corresponding score, and the total score was mapped to the probability of ONFH, illustrating a potential approach for clinical application.

Calibration curves (Figure 5b) demonstrated good agreement between estimated and observed probabilities in both the training and internal validation cohorts, indicating satisfactory calibration performance within this exploratory study. In the external validation cohort, slight deviations from the ideal reference line were observed.

Decision curve analysis (Figure 5c) showed that in the training cohort, the combined model, radiomics model, and clinical model had comparable net benefit across most threshold probabilities. However, in the internal and external validation cohorts, the combined model consistently demonstrated lower net benefit than the radiomics model and was also inferior to the clinical model across a wide range of thresholds (e.g., at threshold 0.2, net benefit: radiomics 0.85 vs. combined 0.80 in internal validation; radiomics 0.85 vs. combined 0.70 in external validation). These findings suggest that adding clinical variables to the radiomics score did not improve clinical utility in independent datasets, and the radiomics model alone provided more stable decision-making benefit.

## 4 Discussion

ONFH represents a serious complication that significantly compromises the quality of life in SLE patients (3–5, 8). Currently, MRI-based radiomics is a feasible method to evaluate bone marrow microenvironment, which has been applied to the classification of osteoporosis and bone tumors (20, 23). In this exploratory study, MRI-based radiomics features derived from remote bone marrow regions (the greater trochanter and ilium) differed significantly between symptomatic SLE patients with and without ONFH. The radiomics model demonstrated stable discriminative performance in both internal and external validation cohorts and provided more consistent clinical utility than the combined model. These findings suggest that quantitative imaging features extracted from remote marrow regions may capture disease-related skeletal alterations associated with ONFH.

A major strength of this study is the inclusion of an independent external validation cohort. The preservation of model performance in this cohort provides preliminary evidence of robustness under similar imaging conditions. However, because all MRI examinations were acquired using scanners from the same vendor, true cross-platform generalizability remains unproven and requires validation across heterogeneous imaging systems.

In this cohort, the majority of SLE patients were female and predominantly of childbearing age, consistent with previous epidemiological studies (37). Triglycerides and HDL-C were identified as significant factors in the clinical model. This finding is consistent with previous studies reporting that higher triglyceride levels are associated with an increased risk of osteonecrosis in SLE populations (38). And, lipid metabolism has been implicated in the pathogenesis of ONFH, with evidence of dyslipidemia, abnormal lipid metabolites, and bone marrow adipocyte remodeling contributing to disease development (39–41). Nevertheless, the clinical model showed limited discriminative performance, reflecting the multifactorial nature of SLE-associated ONFH and highlighting the need for imaging-based biomarkers that may capture biological information beyond conventional clinical variables.

At present, there are relatively few studies using imaging methods to predict ONFH, and all of these studies predict it by analyzing the imaging changes caused by the blood flow and metabolic changes within the femoral head (13–16, 42). And Most studies focus on using radiomics or deep learning to analyze the femoral head, aiming to achieve early diagnosis of femoral head osteonecrosis (ONFH) or to predict and estimate bone collapse (27–29). In contrast, this study demonstrates that radiomics features derived from the greater trochanter and ilium can effectively diagnose ONFH in SLE patients. These findings suggest that ONFH in SLE may involve systemic alterations in the bone marrow microenvironment, rather than being solely a localized process. This interpretation is consistent with prior evidence indicating that osteonecrosis involves systemic alterations in bone metabolism and microstructure. The study of Calder et al. (35) found extensive osteonecrosis of the proximal femur within 4 cm below the lesser trochanter in the ONFH group. Subsequently, Markus Tingart et al. found that the expression of BMP-2 gene, the number of osteoblasts/osteoblasts, and the microstructure parameters of bone trabeculae changed in the femoral metaphysis of ONFH patients. These data further indicate that when femoral head necrosis occurs, changes in bone microstructure can extends to the femoral metaphysis (43). It was found that iliac bone biopsy specimens from patients with aseptic osteonecrosis showed significantly reduced rates of osteoblast attachment and bone formation, suggesting that patients with aseptic osteonecrosis had an in apparent metabolic disorder (44). The finding of low bone marrow activity in the iliac crest by Hernigou and Beaujean (36) suggests that the abnormal bone marrow activity associated with osteonecrosis of the hip may be systemic. Therefore, selecting the greater trochanter and ilium as regions of interest is biologically plausible, as radiomics features from these sites may reflect both systemic and localized bone remodeling processes associated with ONFH.

Radiomics features from these regions may capture early microenvironmental alterations associated with ONFH. These alterations may involve immune-mediated inflammation, lipid metabolism dysregulation, and microvascular impairment, which together contribute to bone marrow remodeling and increased susceptibility to osteonecrosis. Although the exact biological correlates of these radiomics features remain unclear, prior studies have suggested that texture-based features may indirectly reflect variations in bone marrow composition, vascularity, and microstructural organization (18–26). Therefore, radiomics may provide a non-invasive approach for characterizing bone marrow alterations associated with ONFH. Future longitudinal studies are needed to determine whether such imaging changes precede clinically apparent ONFH and may contribute to risk stratification in SLE patients. Taken together, our findings suggest an association between remote bone marrow radiomics features and ONFH in SLE. Whether these imaging differences reflect systemic bone marrow microenvironment alterations remains uncertain and requires histological, molecular, and prospective validation.

In the research of radiomics, there are more challenges and problems to face. Identification of optimal machine-learning methods for radiomic applications is a crucial step toward stable and clinically relevant radiomic biomarkers (45). Therefore, different feature selection methods and classification methods may be combined to form a simple, efficient and stable machine learning classifier (46). In order to maximize the accuracy of the selected radiomics features, LASSO, RF and SVM-RFE were applied in our study to perform key feature selection on the extracted features and generate radiomics scores.

Despite the inclusion of an independent external validation cohort, which strengthens the generalizability of the findings, several limitations should be acknowledged. First, due to the retrospective design, we could not collect complete data on glucocorticoid dosage, pulse therapy, or SLE disease activity scores (e.g., SLEDAI). The clinical model therefore included only a limited set of variables. Second, the temporal split between training and internal validation cohorts may introduce bias, as we did not formally evaluate protocol consistency over time. Third, the external validation cohort was small (*n* = 44, with 19 ONFH cases), limiting statistical power; the lack of a statistically significant difference between the combined and clinical models should be interpreted cautiously, and the numerically higher AUC of the combined model requires confirmation in larger studies. Fourth, shape features were excluded *a priori* due to ROI size and geometry variability, and we did not compare model performance with vs. without them. Fifth, we did not compare different feature selection strategies (e.g., union of algorithms) or perform nested cross-validation. Six, all MRI scans were from the same vendor, and cross-platform generalizability remains unproven. Seventh, the ROI definition for the ilium, although reproducible, lacks literature-based standardization. Finally, the biological interpretations of radiomics features remain speculative without histological or molecular validation.

Our findings raise the hypothesis that ONFH in SLE may involve systemic bone marrow microenvironment alterations; however, causality cannot be established from this correlational radiomics study. Future histologic and molecular studies are needed to test this hypothesis.

## 5 Conclusion

MRI-based radiomics features derived from the greater trochanter and ilium differed significantly between symptomatic SLE patients with and without ONFH. These findings suggest that radiomics analysis of remote bone marrow regions may provide a potential imaging biomarker for ONFH identification in SLE. The independent radiomics model demonstrated stable discriminative performance and favorable clinical net benefit in both internal and external validation cohorts. Further prospective multicenter studies are warranted to validate these findings and explore their potential clinical applications.

## Data Availability

The raw data supporting the conclusions of this article will be made available by the authors, without undue reservation.
