# Supplementary material for: Non-femoral head MRI radiomics as a potential imaging biomarker for identifying osteonecrosis of the femoral head in systemic lupus erythematosus patients
Source: Front Med (Lausanne). 2026 Jul 22;13:1889377. doi: 10.3389/fmed.2026.1889377 (PMC13437393; doi:10.3389/fmed.2026.1889377)
Supplement: Supplementary file 1 [file Supplementary_File_1.docx]

**Non-femoral head MRI radiomics as a potential imaging biomarker for identifying osteonecrosis of the femoral head in systemic lupus erythematosus patients**

**Supplementary Table S1: The final important radiological features**

| **ROI** | **Final Importance Radiomic Features** | **Odds Ratio**  **(OR)** | **Confidence Interval**  **(CI)** | **P value** |
| --- | --- | --- | --- | --- |
| **Greater trochanter of the femur** | **A-wavelet.LLLfirstorder_MeanAbsoluteDeviation** | 0.917 | 0.841-0.999 | 0.050 |
|  | **A-wavelet.HHH_firstorder_Mean** | 0.868 | 0.792-0.952 | 0.004 |
|  | **A-wavelet.HHL_glcm_DifferenceVariance** | 0.886 | 0.815-0.962 | 0.005 |
| **Ilium** | **B-original_glr1m_ShortRunLowGrayLevelEmphasis** | 1.092 | 0.998-1.195 | 0.058 |
|  | **B-wavelet.HHL_firstorder_Skewness** | 0.885 | 0.808-0.970 | 0.011 |
|  | **B-wavelet.HHH_glszm_GrayLevelNonUniformity** | 1.123 | 1.033-1.222 | 0.008 |

**Supplementary Figure S1:Distribution of ICC values for radiomics features from (A) the ilium and (B) the greater trochanter.**

**A:the ilium**

**B:the greater trochanter**

**The X‑axis represents ICC values, the Y‑axis represents the number of features, and the red dashed line indicates the ICC threshold of 0.85. Features with ICC > 0.85 were retained for subsequent analysis. After filtering, 560 features from the ilium and 499 features from the greater trochanter were retained.**
